# Hyperhomocysteinemia Causes Chorioretinal Angiogenesis with Placental Growth Factor Upregulation

**DOI:** 10.1038/s41598-018-34187-z

**Published:** 2018-10-25

**Authors:** Yih-Jing Lee, Chia-Ying Ke, Ni Tien, Po-Kang Lin

**Affiliations:** 1 School of Medicine, Fu-Jen Catholic University, Hsinchuang, New Taipei City, 24205 Taiwan; 2 Department of Ophthalmology, School of Medicine, National Yang-Ming University, Taipei, 11221 Taiwan; 3 Department of Ophthalmology, Taipei Veterans General Hospital, Taipei, 11217 Taiwan

## Abstract

Hyperhomocysteinemia is a risk factor for atherosclerosis, which may also be associated with retinal vascular disease, diabetic retinopathy, retinal vein occlusion, and glaucoma. For this study, we established a hyperhomocysteinemia animal model to explore homocysteine (hcy)-related choroidal angiogenesis and possible related factors. We injected Sprague Dawley (SD) rats with different concentrations of hcy and performed color fundus imaging, fluorescein angiography, image-guided optical coherence tomography, and retinal histology to observe the retinal and choroidal changes. Subsequently, we observed prominent choroidal vasculature with congested and tortuous retinal and choroidal vessels in fundus angiographies of the hyperhomocysteinemia animal model. In the histological study, the choroidal capillaries proliferated in the hcy-treated eyes, mimicking choroidal neovascularization. Disrupted retinal pigment epithelium (RPE), abnormal branching vascular network (BVN), and polyp-like structures were also observed in the hcy-treated eyes. Furthermore, we found that placental growth factor (PlGF), but not vascular epithelial growth factor (VEGF), was the key mediating factor of this phenomenon. Our findings suggest that hyperhomocysteinemia might cause choroidal angiogenesis.

## Introduction

High homocysteine (hcy) levels in the blood are thought to be associated with coronary artery disease, atherosclerotic diseases^1,2^, and neural degenerative diseases^3^, including Alzheimer’s dementia^4,5^ and Parkinson’s disease^6^. Similar to neural degeneration, several retinal disorders have been associated with high levels of hcy. High plasma hcy is a risk factor for retinal vascular occlusion^7,8^, which may be improved by the intake of folate, vitamins B6, and B12 supplements^8^. This condition, hyperhomocysteinemia, is also associated with ocular diseases such as diabetic retinopathy^9^, glaucoma^10,11^ and age-related macular degeneration (AMD)^12^. Hcy is usually formed when methionine is metabolized. It can be reprocessed into cysteine by cystathionine-beta-synthase or into methionine by methylene-tetrahydrofolate reductase (MTHFR)^13^. It is therefore patients with MTHFR mutation show hyperhomocysteinemia with different vascular disorders^13^. Apart from MTHFR defects, hyperhomocysteinemia may be possibly caused by diseases such as nephropathy^14^, psoriasis^15^, hypothyroidism^16^, diet problems such as vitamin B12 deficiency, folate deficiency, alcoholism, high intake of methionine^17^ and medication such as nitrous oxide inhalation^18,19^. However, the association between hcy and choroidal diseases is not widely reported.

In developed countries, AMD accounts for more than 50% of vision loss in the elderly population. AMD can be categorized into dry and wet types. The wet type of AMD is associated with macular choroidal neovascularization (CNV), exudation, and hemorrhage^20^ and affected patients may progressively develop metamorphopsia, central scotoma, or vision loss. Freund *et al*. proposed a more refined AMD classification into three types of neovascularization^21,22^. Type I neovascularization is characterized by vessels proliferating beneath the retinal pigment epithelium (RPE), typically presenting as a fairly mature neovascularization that may have an incomplete response to anti-vascular endothelial growth factor (VEGF) therapy^22^. Polypoidal choroidal vasculopathy (PCV) is included in this classification as a variant of type 1 neovascularization. Conversely, type II neovascularization is characterized by the proliferation of neovascular tissue above the RPE, in the subretinal space, and it may have a complete response to anti-VEGF therapy^22^. Type III neovascularization is an intra-retinal neovascularization, also known as retinal angiomatous proliferation^23^. Although several effective therapies have been reported for these diseases, the possible mechanisms involved in these CNV diseases still require clarification.

Growth factors from the VEGF family control angiogenesis and increase vascular permeability in a number of eye diseases, including diabetic retinopathy and AMD^24^. Placental growth factor (PlGF) is also a member of the VEGF family. Both VEGF and PlGF play important roles in vessel sprouting and proliferation in the chorioretinal tissue^25^. Moreover, Klettner *et al*. reported that VEGF and PlGF were constitutively secreted and regulated by the RPE/choroid complex, with PlGF secreting mainly by the choroid^26^. VEGF receptor-1 (VEGFR-1) is a receptor for both PlGF and VEGF-A^24^. VEGFR-1 is reported to exist in pericytes and vascular smooth muscle cells rather than endothelial cells, suggesting the involvement of pericytes in the early phases of angiogenesis^24^. In addition, Inoue *et al*. reported that PlGF has a protective effect on retinal neurons and possibly other retinal cells^27^. Huo *et al*. discovered that inhibiting both VEGF and PlGF expression reduced neovascularization in a mouse model of laser-induced CNV^28^. All these studies have demonstrated that VEGF and PlGF play different roles in neovascularization development.

We previously reported that plasma hcy levels were significantly higher in patients with PCV than in those without PCV^29^, and we found that choroidal angiogenesis was facilitated by hcy in a choroidal capillary sprouting model^30^. Therefore, we assumed that hyperhomocysteinemia may play a role in type I CNV development, including PCV, possibly via an atherosclerosis pathway. In the current study, we investigated the possible characteristics of retinal cellular changes and choroidal vasculopathy in a hyperhomocysteinemia animal model.

## Results

**Figure 1:**
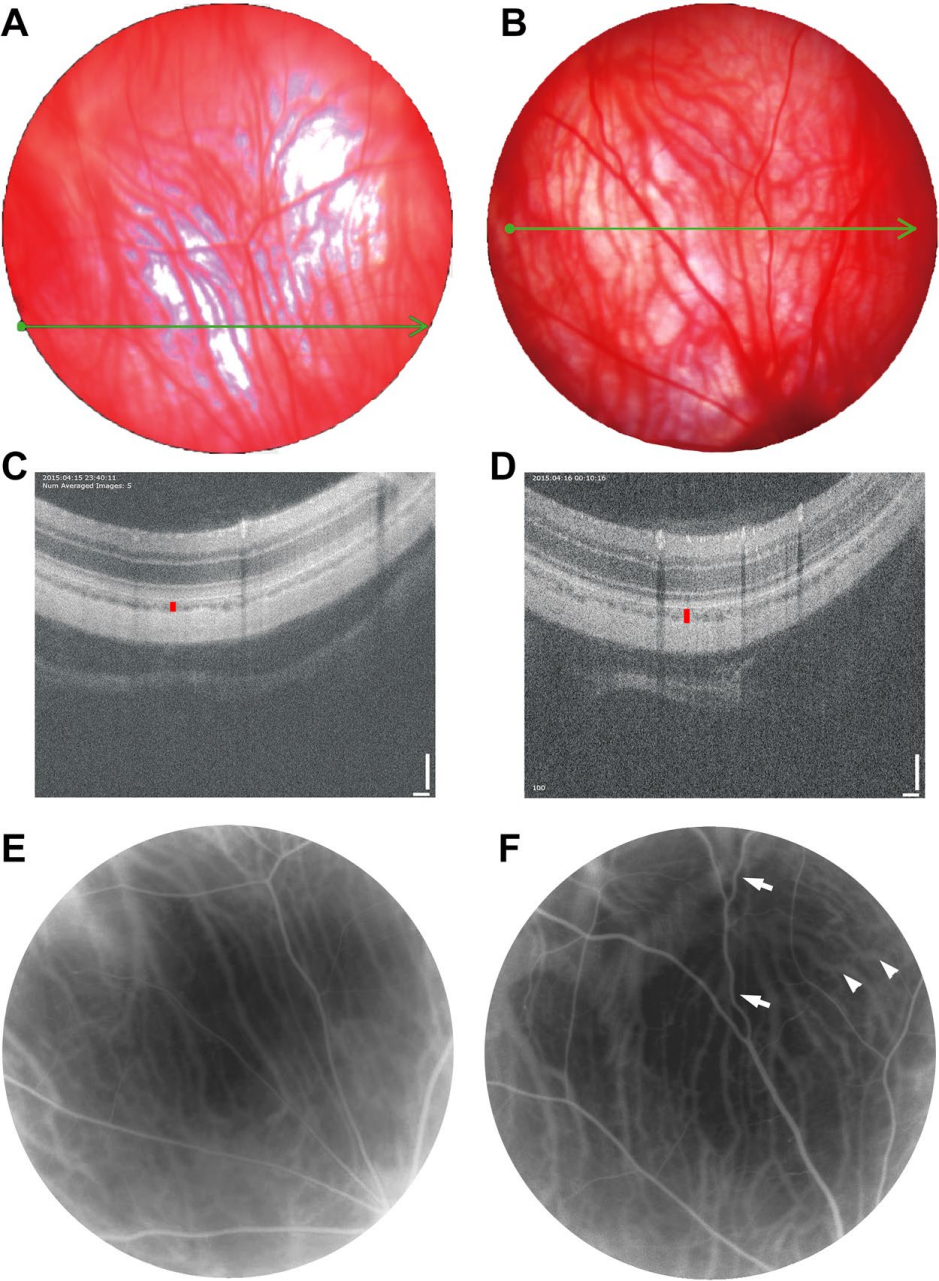
Results of color fundus images, OCT, and FAG of the control and 100 mg/kg hcy-treated animals. The color fundus images of the control and hcy-treated eyes are presented in (**A**,**B**), respectively. The green lines in (**A**,**B**) indicate the location of the image-guided OCT present in (**C**,**D**) for the control and hcy-treated animals, respectively. FAG results of the control (**E**) and hcy-treated animals (**F**) are also presented. We noted significant congestion and tortuosity of the retinal (arrows) and choroidal (arrowheads) vasculatures. OCT revealed that choroid layer thickness increased in the hcy-treated eyes (vertical red line in (**D**), 39 μm) compared with the control eyes (vertical red line in (**C**), 26 μm).

We obtained color fundus images, image-guided optical coherence tomography (OCT), and fluorescein angiography (FAG) results from hyperhomocysteinemia animals and examined the control animals to observe the chorioretinal vessel changes (Fig. 1). In the animals treated with 100 mg/kg hcy, we observed a dense and curly vessel distribution in the choroid layer of the fundus image (Fig. 1B) compared with the control group (Fig. 1A). Image-guided OCT results showed choroidal layer thickening (39 μm in Fig. 1D) compared with the control group (26 μm in Fig. 1C), and FAG results showed significant congestion and tortuosity of the retinal and choroidal vasculature (Fig. 1F) compared with the control group (Fig. 1E). A summary of choroidal thickness in OCT study was shown in Table 1. An increase of mean choroidal thickness in the 100 mg/kg hcy-treated group was seen, but there was no statistical difference between groups.

**Table 1:** Summary of choroidal thickness in OCT study.

Hcy (mg/kg)	Sample Size	Choroidal thickness (um) Mean ± SEM
0	4	27.9 ± 0.30
30	4	28.4 ± 1.68
60	4	28.6 ± 1.93
100	4	31.6 ± 4.85

Choroidal thickness was measured from OCT images of each animal/eye. An increase of mean choroidal thickness in the 100 mg/kg hcy-treated group was seen, but there was no statistical significance between groups. One-way ANOVA was used to analyze the thickness, in which there was no significant difference between groups.

**Figure 2:**
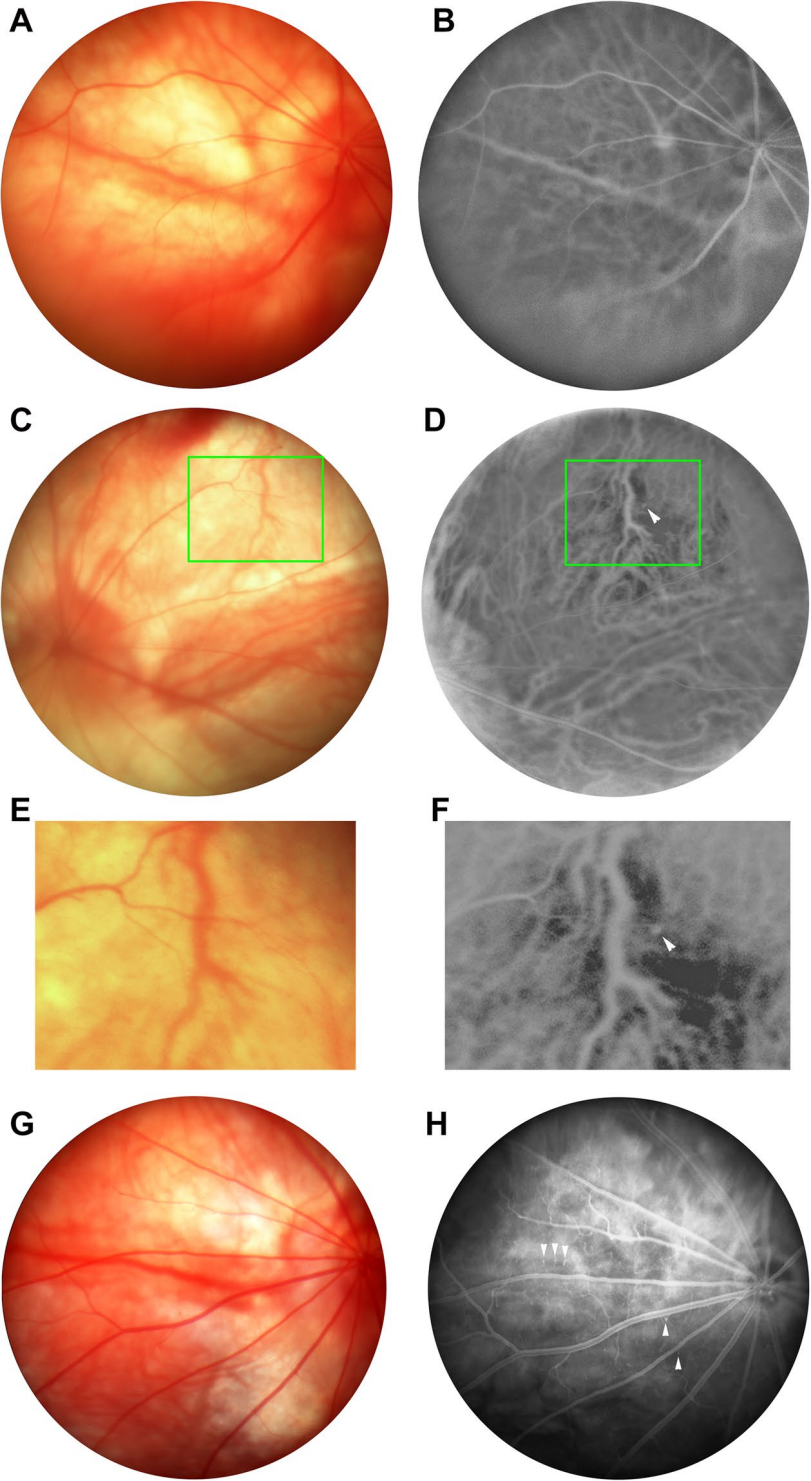
Color fundus images and FAG results of control and hcy-treated animals. The color fundus images of the control and hcy-treated eyes are presented in (**A**,**C**,**E**,**G**), and the FAG results of the control and hcy-treated eyes are presented in (**B**,**D**,**F**,**H**). (**A**,**B**) are from control eyes; (**C**–**F**) are from 30-mg/kg-hcy treated eyes; (**G**,**H**) are from 60 mg/kg-hcy treated eyes. (**E**,**F**) are large-scale representations of the boxes in (**C**,**D**). Arrowheads in (**D**,**F**,**H**) indicate polyps in the choroid vessels on FAG of the hcy-treated animals.

Distributions of the retinal and choroidal vessels in animals treated with 30 mg/kg hcy and control animals were also evaluated on color fundus photography (for retinal vessels) and FAG (for both retinal and choroidal vessels). Figure 2 demonstrates the chorioretinal vasculature becoming more prominent and tortuous (Fig. 2C) in the 30 mg/kg hcy-treated animals than in control animals (Fig. 2A). FAG imaging of the 30 mg/kg (Fig. 2D,F) and 60 mg/kg (Fig. 2H) hcy-treated animals identified polyps in the choroid vessels but not in the retinal vessels (arrowhead in Figs. 2D,F,H). With light-pigmented RPE, FAG can depict the choroidal vessels of Sprague Dawley (SD) rats without the need for indocyanine green angiography. Visibility of this polyp only on FAG (Fig. 2D,F) but not on color fundus images (Fig. 2C,E) of the same SD rat proves its existence in the choroidal layer of the hcy-treated animals. Furthermore, we observed that the polyp was beneath a crossing retinal vessel and located in the same level of nearby choroidal vessels (Fig. 2F). The summary for FAG study was presented in Table 2, which showed vessel tortuosity and statements for FAG features from control, 30, 60, 100 mg/kg hcy animals. The number of vessel tortuosity more than 1.33 was count in each FAG image and list in Table 2. The original FAG images studied for Table 2 were presented in Supplementary Fig. 1. There were notable tortuous retinal and choroidal vessels, congestive vessels, and polyps in hcy-treated animals. The result suggests that hyperhomocysteinemia may induce polyps in the choroidal layer, which is similar to the features of PCV.

**Table 2:** Summary of FAG findings in different animals.

Animal/Eye	Hcy mg/kg	Number of vessel tortuosity >1.33	Mean ± SEM for number of tortuosity >1.33	FAG features
Ctl_56R	0	7	7.0 ± 1.7	Normal retinal and choroidal vessels, no congestive or engorged vessel seen.
Ctl_65L	0	10		Normal retinal and choroidal vessels, no congestive or engorged vessel seen.
Ctl_68R	0	4		Normal retinal and choroidal vessels, no congestive or engorged vessel seen.
30_57R	30	15	11.3 ± 1.9	Congestive choroidal veins, tortuous choroidal vessel, and polyps
30_57L	30	9		Congestive choroidal veins, vessel engorged in central choroid.
30_63R	30	10		Congestive choroidal veins, engorged choroidal veins, and tortuous choroidal vessels.
60_66R	60	9	11.7 ± 2.2	Tortuous retinal vessel, tortuous choroidal vessels, and congestive choroidal veins
60_66L	60	10		Tortuous retinal vessel, tortuous choroidal vessels, and congestive choroidal veins
60_69L	60	16		Tortuous choroidal vessels, and several polyps
100_61L	100	11	12.3 ± 0.7	Congestive choroidal veins, and tortuous choroidal vessels.
100_67L	100	13		Tortuous choroidal vessels, and cluster convoluted choroidal vessels
100_67R	100	13		Congestive retinal veins, engorged choroidal veins, and tortuous choroidal vessels.

The tortuosity of retinal and choroidal vessels were measured via MatLab with ARIA program. Findings from FAG images of each animal/eye are stated in the FAG features. One-way ANOVA was used to analyze the number of vessel tortuosity >1.33 data, in which there were not significant difference between groups.

**Figure 3:**
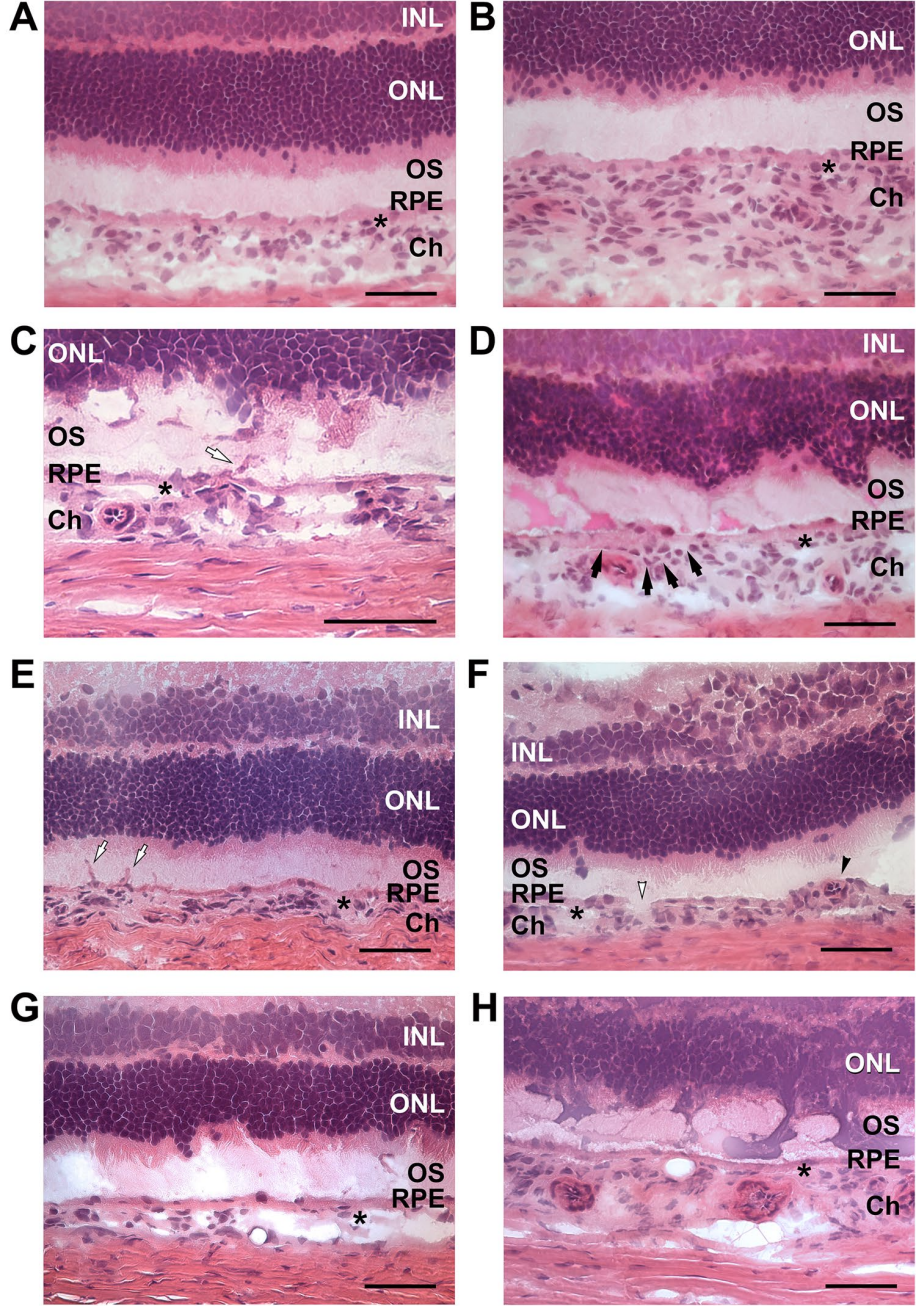
Microscopy images of the retinas of animals treated with different concentrations of hcy or control animals. Outer nuclear layer (ONL), inner nuclear layer (INL), outer segment (OS), choroid (Ch) and RPE were demonstrated in the images. Star signs indicate the Bruch’s membrane in images. Image (**A**) is from the retina of an animal in the control group. Image (**B**) is from an animal treated with 30 mg/kg hcy for 9 days, showing choroidal thickening. Image (**C**) is from an animal treated with 10 mg/kg hcy for 9 days, showing choroidal cell proliferation and neovascularization (white arrow). Images (**D**,**E**) are of retinas treated with 100 mg/kg hcy for 9 days, showing intra-BM cell proliferation (black arrows in (**D**)) and neovascularization (white arrow in (**E**)). Image (**F**) is from 90 days of recovery after the 30 mg/kg 9-day hcy treatment, demonstrating abnormal BVN (black arrowhead) and RPE disruption (white arrowhead). Images (**G**,**H**) are from animals treated with 60 and 30 mg/kg hcy, respectively, showing polyps in different layers of the choroid. Scale bar = 50 µm.

We investigated the histological changes in the hcy-treated and control retinas using hematoxylin and eosin (H&E) staining and optical microscopy (Fig. 3). An evenly distributed outer nuclear layer (ONL), inner nuclear layer (INL), outer segment (OS), choroid (Ch) and RPE was demonstrated with normal endothelial cells located in the choroid vessels in a chorioretinal section of a control animal (Fig. 3A). Choroidal thickening was detected (Fig. 3B) in the retinas of animals treated with 30 mg/kg hcy for 9 days, similar to pachychoroid in PCV patients. In addition, choroidal cell proliferation and neovascularization were identified (white arrow in Fig. 3C) in animals treated with 10 mg/kg hcy for 9 days. Intra-Bruch’s membrane (BM) cell proliferation in a curved pattern (Fig. 3D) and neovascularization (white arrow in Fig. 3E) were observed in the retinas of animals treated with 100 mg/kg hcy for 9 days (Fig. 3D,E). We observed an abnormal branching vascular network (BVN) in an ovoid pattern (black arrowhead) and RPE disruption (white arrowhead) in the retinas of animals after recovering for 90 days following a 30 mg/kg hcy treatment for 9 days (Fig. 3F). Polyps in different locations of the choroid were also observed in animals treated with 60 and 30 mg/kg hcy (Fig. 3G,H), corresponding to the characteristics of PCV in the hyperhomocysteinemia animal model. Low-power microscopic images presenting retinal sections from different hcy-treated and control groups were presented in Supplementary Fig. 2. Table 3 presents a summary of the histological characteristics of the animals treated with different concentrations of hcy in addition to details of the characteristics and the number of the animals. These histological studies indicate that hyperhomocysteinemia may lead to vascular endothelial cell proliferation and possibly neovascularization of the choroid.

**Table 3:** Histological characteristics of choroid and retina of the hyperhomocysteinemia animal models.

Dose (mg/kg)	Number of animals with the characters/number of animals observed	Histological characters
0	4/4	Normal retina, RPE and choroid
10	1/4	Choroidal cell proliferation
30	2/4	Choroidal thickening, intra-BM cell proliferation, neovascularization, and polyps
60	3/4	Polyps, neovascularization, and choroidal cell proliferation
100	3/4	Choroidal cell proliferation, intra-BM cell proliferation, and neovascularization
30 (90 days^a^)	2/2	Abnormal BVN, and RPE disruption

^a^Note: follow up for 90 days after injection.

**Figure 4:**
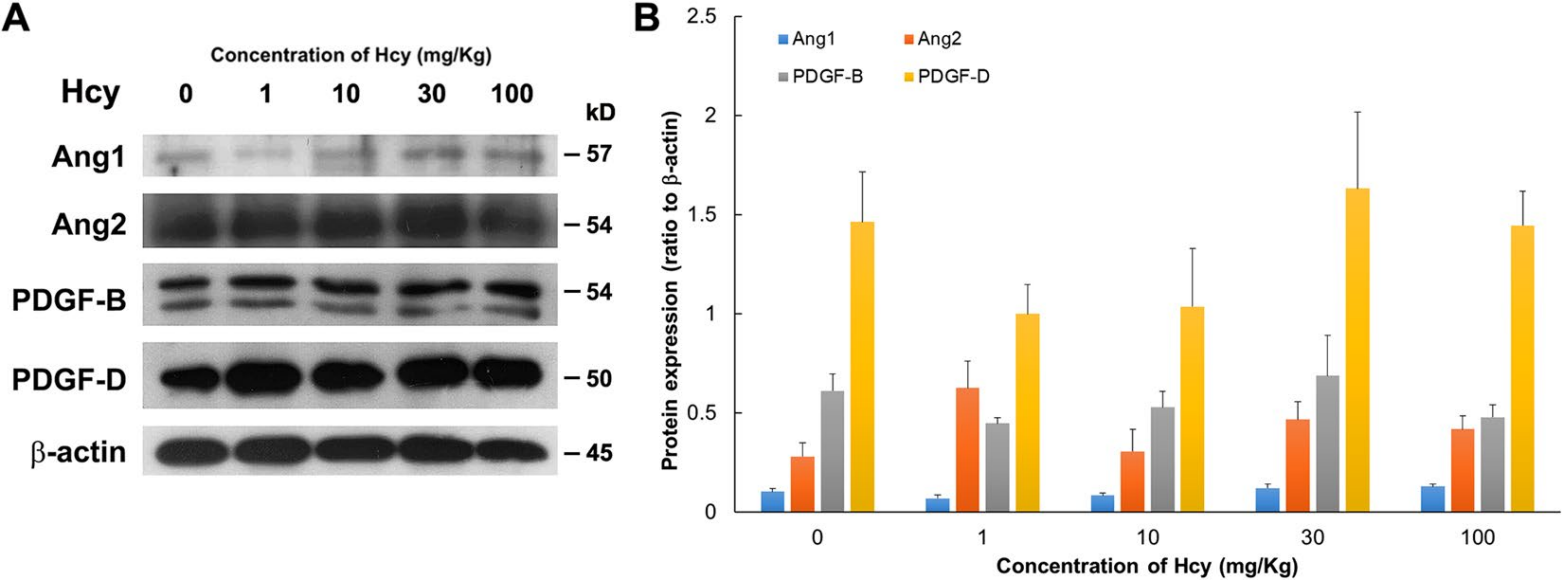
Ang1, Ang2, PDGF-B, and PDGF-D protein expression was present in the retinas of the different animals. (**A**) Western blotting of retinas with Ang1, Ang2, PDGF-B, and PDGF-D expression from the control animals and the animals treated with 1, 10, 30, or 100 mg/kg hcy. (**B**) Statistical analyses of Ang1, Ang2, PDGF-B, and PDGF-D protein expression in the retinas from the control and hcy-treated animals. The data are presented as mean values, with error bars representing the standard error of the mean (SEM). One-way ANOVA was used to analyze the data. N = 5 in each group. Details regarding the data are shown in Supplementary Table 1, and full-length blots are presented in Supplementary Fig. 3.

**Figure 5:**
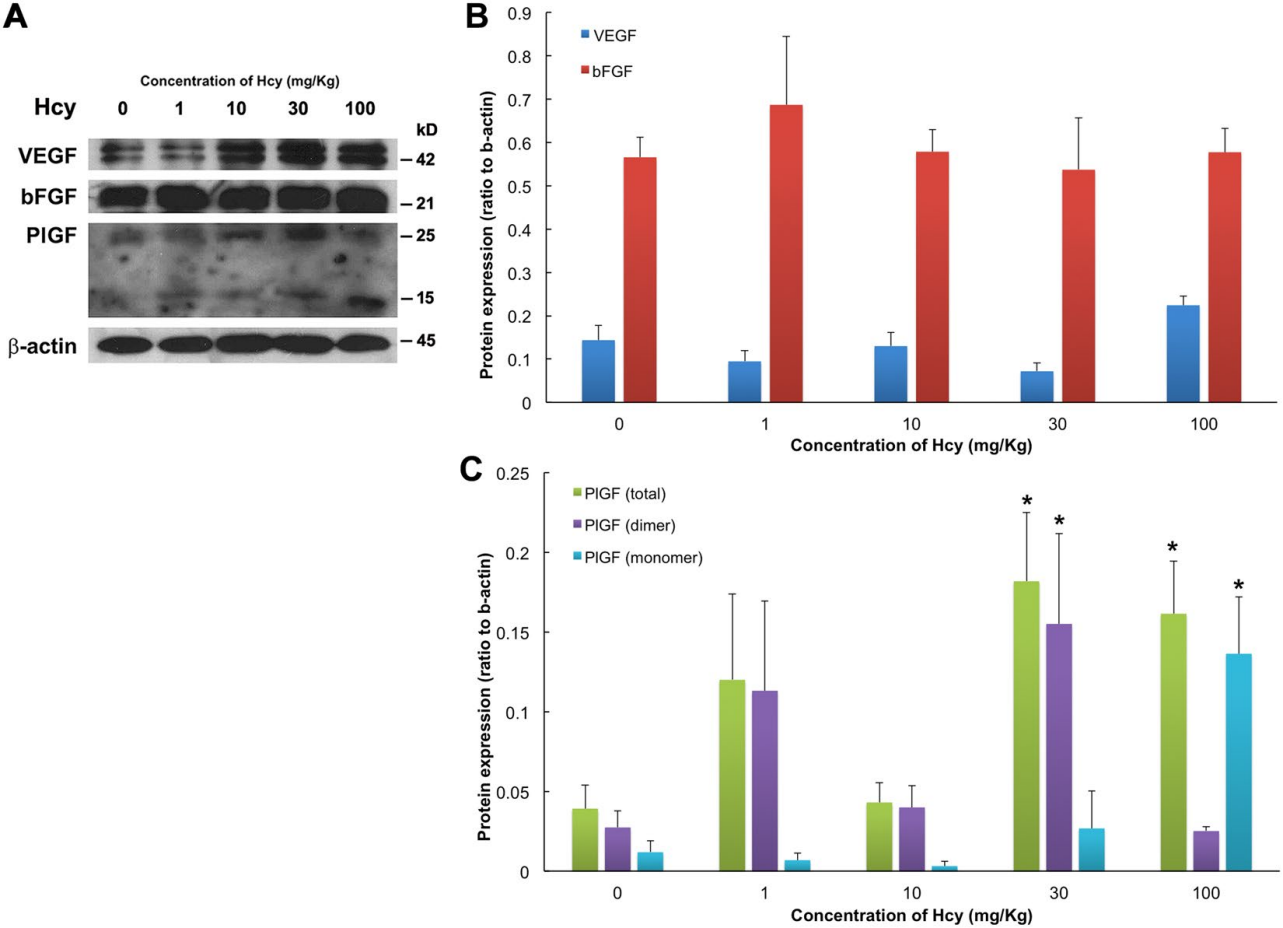
VEGF, bFGF, and PlGF protein expression was present in the retinas of the different animals. (**A**) Western blotting from retinas with VEGF, bFGF, and PlGF expression collected from the control animals and the animals treated with 1, 10, 30, or 100 mg/kg hcy. PlGF dimer-form is detected at 25kD, PlGF monomer-form is detected at 15kD, and total PlGF combines expressions from both dimer- and monomer-forms. (**B**,**C**) Statistical analyses of VEGF, bFGF, and PlGF protein expression in the retinas of control and hcy-treated animals. The data are shown as mean values, with error bars representing the SEMs. One-way ANOVA with Dunnett’s test for multiple comparisons was used to analyze the data. N = 5 in each group. ^*^Indicates *P* < 0.05 compared with the control group. Details regarding the data and *P* values are shown in Supplementary Table 1, and full-length blots are presented in Supplementary Fig. 3.

**Figure 6:**
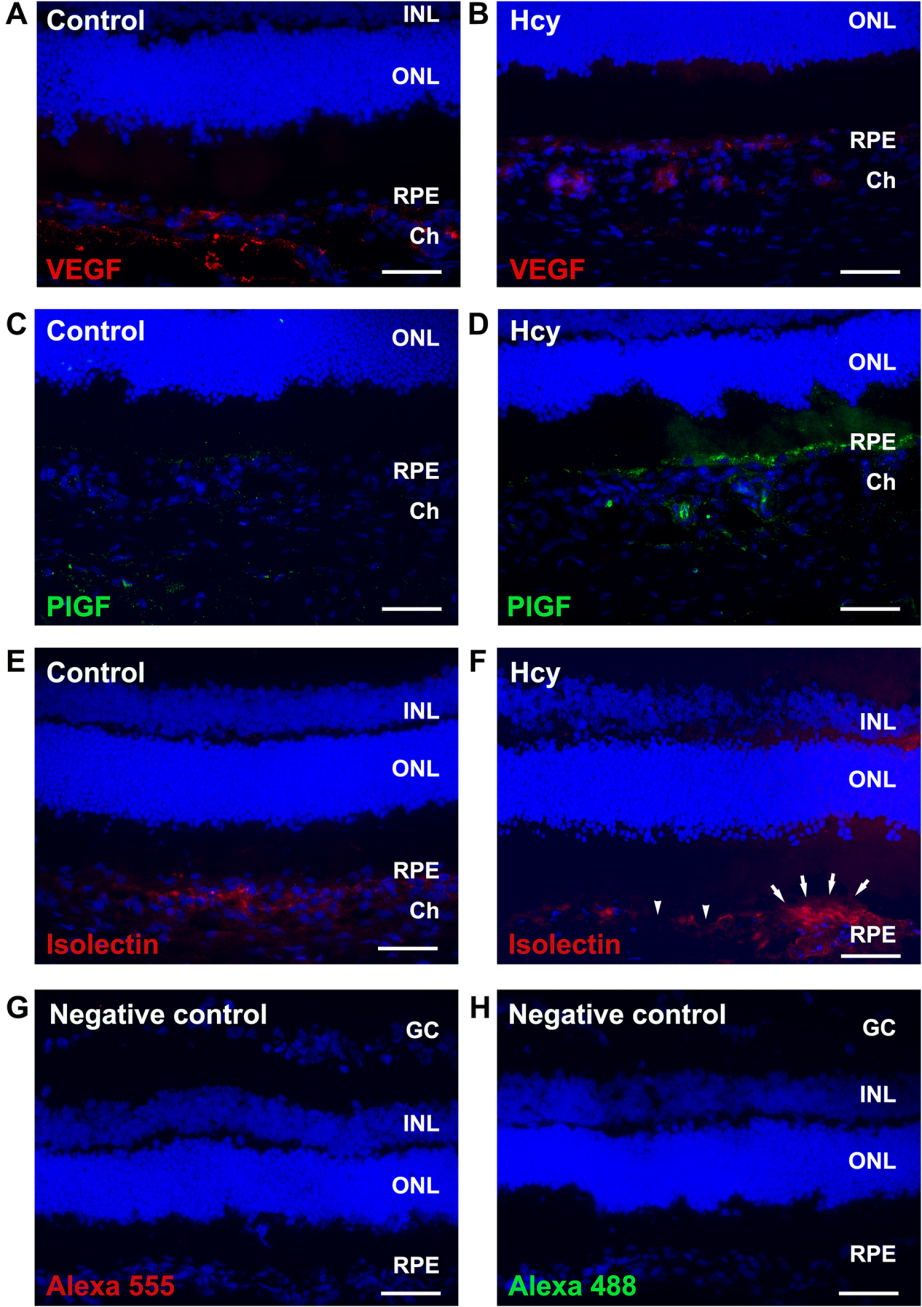
Immunofluorescence staining of the chorioretinas treated or not treated with hcy. Images (**A**,**C**,**E**) are of the control animals. Images (**B**,**D**,**F**) are of the 30 mg/kg hcy-treated animals. Images (**A**,**B**) show immunoreactions of VEGF (red, Alexa 555), while images (**C**,**D**) show immunoreactions of PlGF (green, Alexa 488). Images (**E**,**F**) show location of choroidal vessels labeled with isolectin IB4 (red). Neovascularization above the RPE area (arrows) and RPE disruption (arrowheads) were noticed in the hcy-treated eye (**F**). Images (**G**,**H**) are negative control staining for Alexa 555 and Alexa 488 without primary antibody. Cell nuclei were stained with DAPI (blue). PlGF was highly expressed only in the RPE and choroid of the hcy-treated eyes. VEGF increased slightly in the RPE and choroid of the hcy-treated eyes. Scale bar = 50 µm.

The protein expression levels of angiopoietin1 (Ang1), angiopoietin2 (Ang2), platelet-derived growth factor B (PDGF-B), platelet-derived growth factor D (PDGF-D), VEGF, basic fibroblast growth factor (bFGF), and PlGF were evaluated in the chorioretinal tissue of different animals using Western blotting, and the results are shown in Figs 4 and 5. We examined the animals from the control, 1, 10, 30, and 100 mg/kg hcy-treated groups and found that PlGF expressions in animals treated with 30 and 100 mg/kg hcy were significantly upregulated compared with that in the control group (by 11.5-fold for PlGF monomer and 4.1-fold for total PlGF in 100 mg/kg hcy group, by 5.7-fold for PlGF dimer and 4.6-fold for total PlGF in 30 mg/kg hcy group; Fig. 5C and Supplementary Table 1), corresponding to increased immunofluorescence staining of the choroid as shown in Fig. 6. The expression of VEGF appeared to increase slightly in the 100 mg/kg hcy-treated animals, but this was not statistically significant (Fig. 5B, *P* = 0.072, compared to control group). The details of statistical analysis are displayed in Supplementary Table 1. In addition, there were no significant changes in the Ang1, Ang2, PDGF-B, and PDGF-D expression levels in these groups (Fig. 4B).

**Figure 7:**
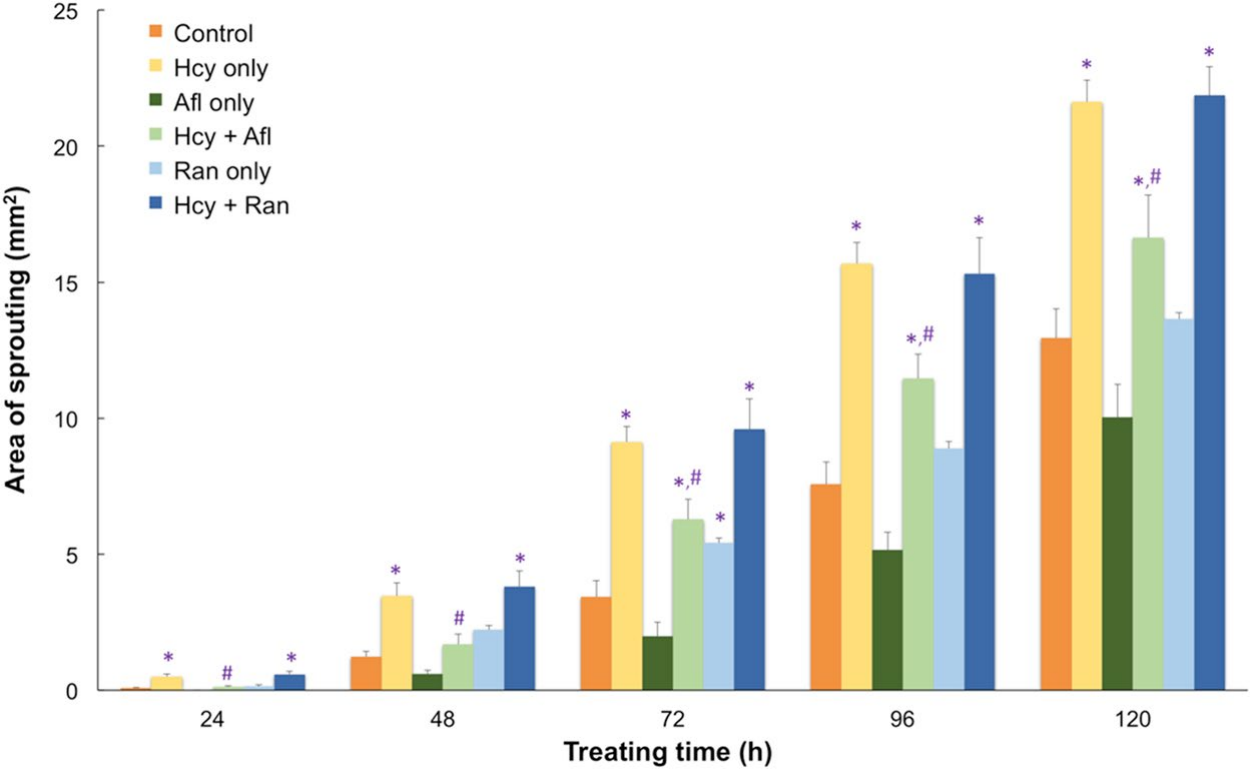
Capillary sprouting areas in the retinal explants treated with hcy and different anti-VEGF medications. The bar chart indicates the capillary sprouting areas from the retinal explants treated with 1 mM hcy, 1 mg/mL aflibercept (Alf), or 0.25 mg/mL ranibizumab (Ran). Data are indicated as mean values with error bars representing the SEMs. One-way ANOVA with LSD multiple comparison was used for data analysis. N = 4–8, *indicates a *P* value < 0.05 compared with the control group, and ^#^indicates a *P* value < 0.05 compared with the hcy-only group.

Using immunofluorescence staining, the retinal sections from the hyperhomocysteinemia animal model were continuously examined for the expression of various growth factors in the retinas (Fig. 6). In animals treated with or without hcy (Fig. 6A,B), VEGF was expressed around the RPE-choroid area of the retinas. In comparison, PlGF was expressed only in the choroid area of the animals treated with hcy (Fig. 6D), but not in the control animals (Fig. 6C), suggesting that PlGF may play a key role in retinal and choroidal angiogenesis induced by hcy. Isolectin IB4 was also used to label the choroidal endothelial cells indicating location of vessels in control and hcy-treated eye (Fig. 6E,F). A neovascularization at the RPE area and RPR disruption were noticed in the hcy-treated eye labeled with isolectin (Fig. 6F). In addition, we used two well-known anti-VEGFs, aflibercept and ranibizumab, subsequently to study possible factors involved in the chorioretinal vascularization with a choroidal capillary sprouting model. Aflibercept and ranibizumab are the two major anti-VEGFs used to treat diabetic retinopathy and neovascular AMD, including PCV^31^. Statistical results of the capillary sprouting area are shown in Fig. 7. In total, 1 mM of hcy and 1 mg/mL of aflibercept or 0.25 mg/mL of ranibizumab was used in this study. Statistical data are presented in Table 4. Addition of aflibercept to the hcy-treated chorioretinal explants inhibited the increase in the capillary sprouting area caused by hcy. However, ranibizumab did not indicate any similar inhibition effect on the hcy-treated preparations. The different effects caused by these two agents indicate that aflibercept may inhibit angiogenesis in the choroid caused by hcy, and this attributed to its unique feature that traps and blocks both VEGF and PlGF. However, ranibizumab blocks VEGF but not PlGF. We assume that PlGF upregulation might play a key role in hcy-induced chorioretinal vascularization. On the basis of the aforementioned results, we have determined that PlGF plays a key role in hcy-induced choroidal angiogenesis.

**Table 4:** Data on the effects of anti-VEGFs on capillary sprouting from the retinal explants.

Group	Treating Time (h)	24	48	72	96	120
Control	N	8	8	8	8	8
	Mean ± SEM	0.08 ± 0.03	1.23 ± 0.20	3.43 ± 0.60	7.57 ± 0.83	12.95 ± 1.06
Hcy only	N	8	8	8	8	8
	Mean ± SEM	0.50 ± 0.09	3.47 ± 0.47	9.13 ± 0.57	15.69 ± 0.77	21.62 ± 0.80
	*P value to control*	*0*.*000*^a^	*0*.*000*^a^	*0*.*000*^a^	*0*.*000*^a^	*0*.*000*^a^
Afl only	N	4	4	4	4	4
	Mean ± SEM	0.00 ± 0.00	0.60 ± 0.13	1.98 ± 0.51	5.15 ± 0.66	10.03 ± 1.20
	*P value to control*	*0*.*468*	*0*.*265*	*0*.*126*	*0*.*056*	*0*.*052*
Hcy + Afl	N	4	4	4	4	4
	Mean ± SEM	0.13 ± 0.04	1.68 ± 0.38	6.28 ± 0.73	11.46 ± 0.89	16.64 ± 1.57
	*P value to control*	*0*.*672*	*0*.*421*	*0*.*004*^a^	*0*.*003*^a^	*0*.*016*^a^
	*P value to hcy only*	*0*.*001*^b^	*0*.*003*^b^	*0*.*004*^b^	*0*.*001*^b^	*0*.*001*^b^
	*P value to Afl only*	*0*.*321*	*0*.*100*	*0*.*000*^c^	*0*.*000*^c^	*0*.*000*^c^
Ran only	N	4	4	4	4	4
	Mean ± SEM	0.14 ± 0.07	2.22 ± 0.15	5.42 ± 0.17	8.88 ± 0.26	13.65 ± 0.24
	*P value to control*	*0*.*611*	*0*.*082*	*0*.*039*^a^	*0*.*293*	*0*.*635*
Hcy + Ran	N	4	4	4	4	4
	Mean ± SEM	0.59 ± 0.10	3.82 ± 0.57	9.59 ± 1.11	15.31 ± 1.33	21.86 ± 1.07
	*P value to control*	*0*.*000*^a^	*0*.*000*^a^	*0*.*000*^a^	*0*.*000*^a^	*0*.*000*^a^
	*P value to hcy only*	*0*.*451*	*0*.*542*	*0*.*620*	*0*.*758*	*0*.*871*
	*P value to Ran only*	*0*.*001*	*0*.*018*	*0*.*000*^d^	*0*.*000*^d^	*0*.*000*^d^

The data below present the capillary sprouting areas (mm^2^). Sample size (N) and mean ± standard error of the mean (SEM) are presented. Afl stands for aflibercept, and Ran stands for ranibizumab. One-way ANOVA with LSD multiple comparison was used for data analysis. ^a^Indicates a *P* value < 0.05 compared with the control group; ^b^indicates a *P* value < 0.05 compared with the hcy-only group; ^c^indicates a *P* value < 0.05 compared with the aflibercept-only group; and ^d^indicates a *P* value < 0.05 compared with the ranibizumab-only group.

## Discussion

Hcy is a sulfur-containing amino acid and metabolite of the essential amino acid methionine existing at a critical biochemical intersection in the methionine cycle^32^. The half-life of hcy in plasma is 223 ± 45 min (around 3.7 h)^33^, which suggests that the amount of hcy would reduce to 1.1% after 24 h. Consequently, we saw the pathological changes of the retina and choroid under high hcy concentrations (10–100 mg/kg) but not under low concentrations (1 mg/kg) in our animal model. A high serum level of hcy signals a breakdown in this vital process, which results in far-reaching biochemical and life consequences^32^. Hyperhomocysteinemia is related to small-vessel disease and atherosclerosis of the brain^2^ and possibly vascular dementia and Alzheimer’s disease^4,5^. Previous studies have also suggested that hcy may be a risk factor for retinal vascular disease or a key factor in inducing retinal ganglion cell apoptosis^8,34,35^. A recent study suggests that hyperhomocysteinemia may possibly induce retinal endothelial cell dysfunction by activating oxidative stress in the endothelial cells^36^. Furthermore, we have reported that hyperhomocysteinemia was identified in PCV patients^29^. In our current study of a hyperhomocysteinemia animal model, choroidal cell proliferation, polyps, BVN, intra-BM cell proliferation, and neovascularization were observed in the microscopic study, suggesting that angiogenesis might progress in the presence of hyperhomocysteinemia. These histological findings were identified in those animals treated with 10, 30, 60, and 100 mg/kg hcy for 9 days, and some of the pathological changes persisted for 90 days following the hcy injection course. These histological findings are similar to the pathological features of type I CNV in humans^22^. Induced hyperhomocysteinemia in animals is similar to hyperhomocysteinemia in patients with PCV^29^. Because PCV is a variant of type I CNV, according to Freund *et al*., when combining these facts, this animal model might mimic human PCV in terms of choroidal angiogenesis^22^. Moreover, a thick choroid, a so-called pachychoroid, is associated with macular disease, e.g., PCV^37^. Pachychoroid is characterized by increased choroidal thickness and dilated choroidal vessels. In our hcy-injected rats, similar pictures were revealed on both OCT and chorioretinal histopathology, which suggests that hyperhomocysteinemia might cause chorioretinal cellular changes.

In wet AMD, upregulation of VEGF expression in the eye was considered to be related to CNV^38,39^. CNV was thought to be a prominent feature of wet AMD^40^, and the suppression of VEGF expression was considered a possible solution for neovascularization in this type of disease^41,42^. Clinically, CNV is commonly treated with an intravitreous injection of anti-VEGF antibody. Hence, we investigated the expression levels of different types of VEGFs in this study, revealing that VEGF was detected in the choroid of the eyes of both the control and hyperhomocysteinemia animals; however, PlGF was detected in the choroid of the eyes of only the hyperhomocysteinemia animals. In this study, aflibercept and ranibizumab were used to investigate the possible mechanisms associated with hcy-induced choroid vessel sprouting. Clinically, aflibercept has potent therapeutic effects on neovascular AMD and retinal vascular diseases^43,44^. With regard to PCV treatment, aflibercept is more effective than ranibizumab^31,45,46^. These two drugs differ in terms of structure and mechanism. Ranibizumab, a monoclonal antibody fragment with a Fab domain of anti-VEGF-A, is designed to better penetrate the retina and choroid, and it only blocks VEGF-A^47^; hence, it only suppresses the activation of VEGFR-1. Aflibercept, a recombinant fusion protein with combined VEGFR-1 and VEGFR-2 binding domains^48^, effectively inhibits the effects of both VEGF-A and PlGF. Our results showed that aflibercept inhibited hcy-evoked capillary sprouting, whereas ranibizumab did not. This suggests PlGF may play a key role in hcy-induced chorioretinal vascularization.

Moreover, we investigated the expression of other angiogenesis-related growth factors, such as Ang1, Ang2, PDGF-B, and PDGF-D, of the retinal and choroidal preparations from both the control and hcy-treated animals. Angiopoietins are proteins involved in vascular development and angiogenesis. For instance, Ang1 promotes endothelial cell sprouting and tube formation^49^, whereas Ang2 regulates angiogenesis and endothelial inflammation^50^. In addition to angiopoietins, PDGFs are important in the development of connecting tissues, including endothelial and epithelial cells^51^. However, expressions of angiopoietins and PDGFs were not found to be altered in the different groups in this study. Our findings suggest that PlGF may be upregulated by hyperhomocysteinemia and may play a role in choroidal vessel proliferation in this animal model. PlGF is a member of the VEGF family but it binds to different VEGF receptor subtypes. Inoue *et al*. reported that PlGF has a protective effect on retinal neurons and possibly other retinal cells^27^. Kowalczuk *et al*. also reported that tortuous and dilated capillaries were observed in a PlGF over-expression animal model^52^, which is similar to our findings in FAG study of the hcy-treated animals. In this hyperhomocysteinemia animal model, we found that the presence of hcy considerably enhanced PlGF expression; PlGF was significantly increased in the RPE and choroid layers, but VEGF was slightly increased; thus, we suggest that PlGF may play a key role in hcy-induced choroidal angiogenesis.

We conclude that hyperhomocysteinemia caused retinal cellular changes and choroidal angiogenesis, which may be related to hcy-induced atherosclerosis. An upregulation of PlGF was also observed in the hyperhomocysteinemia animals. Our hyperhomocysteinemia animal model demonstrates pathological features similar to type I CNV, which corresponds to hyperhomocysteinemia in patients with clinical PCV.

## Materials and Methods

### Animal Model of Hyperhomocysteinemia

SD rats (250–300 g, male, from BioLASCO Technology, Taipei, Taiwan) were maintained in a normal light environment of 100 lux. All animal protocols were conducted in accordance with the Statement for the Use of Animals in Ophthalmic and Vision Research outlined by the Association for Research in Vision and Ophthalmology and approved by the Institutional Animal Care and Use Committee of Fu-Jen Catholic University and Taipei Veterans General Hospital. The hyperhomocysteinemia animal model was generated by the intravenous administration of various doses of hcy through the tail veins of the animals. The animals received 1, 10, 30, 60, or 100 mg/kg of hcy once a day for 9 consecutive days. In the control group, normal saline (0.9%) was administered instead of hcy. The animals were euthanized 1 day after completion of the injection course, and both eyes were collected for histological and immunohistochemical examinations as well as a protein expression assay. Several rats from the 30 mg/kg group were kept for another 90 days of recovery after the 9-day injection course and were subsequently euthanized, and the eyes were collected for histological examination.

### Fundus Images

One day after completion of the intravenous injection course, fundus images were obtained in both groups to compare the chorioretinal vascular changes. The procedure for obtaining fundus images is briefly described as follows. During the examinations, the animals were sedated with Zoletil (40 mg/kg; Virbac SA, Carros, France). Their pupils were dilated with 1.0% tropicamide (Alcon, Fort Worth, TX, USA), and the corneas were locally anesthetized with 0.5% proparacaine (Alcon). A retinal imaging microscope system (Micron IV, Phoenix Research Labs, Pleasanton, CA, USA) was utilized for color fundus photography, FAG, and image-guided OCT. The tortuosity of retinal and choroidal vessels in FAG images were analyzed via MatLab (The MathWorks, Inc. Natick, MA, USA) with ARIA (Automated Retinal Image Analyzer, Copyright 2011 Peter Bankhead) implements the vessel detection and tortuosity measurement^53^, in which vessel tortuosity measure is defined as the vessel segment length divided by the Euclidean distance between the vessel segment end points. Number of vessel tortuosity more than 1.33 was counted and presented in Table 2.

### Histological Analysis of the Retina and Choroid

The histologies of the retinal and choroidal vessels were examined using H&E staining. The eyes obtained from rats treated with different preparations were embedded in a tissue-freezing medium and frozen at −80 °C. The eyes were immersed in a fixative solution containing 4% paraformaldehyde in phosphate-buffered saline (pH 7.4) and cut into 5-μm-thick sections using a cryostat (CM3050 S, Leica, Wetzlar, Germany). These sections were stained with hematoxylin (Muto Pure Chemicals Co., Ltd., Tokyo, Japan) and eosin Y (Thermo Fisher Scientific, Kalamazoo, MI, USA) and mounted with HistoMount (Thermo Fisher Scientific) to assess the retinal and choroidal histology. Morphological changes were observed using an upright microscope (DM2500, Leica) and a digital camera system (DFC420, Leica).

### Immunoblotting analysis

The protein expressions of β-actin, VEGF, bFGF, Ang1, Ang2, PDGF-B, PDGF-D, and PlGF were studied using Western blotting. The primary antibodies used in this study were anti-β-actin (4970, Cell Signaling Technology, Danvers, MA, USA), anti-VEGF (sc-152, Santa Cruz Biotechnology, Dallas, TX, USA), anti-bFGF (sc-79, Santa Cruz Biotechnology, Santa Cruz, CA, USA), anti-Ang1 (ab95230, Abcam, Cambridge, MA, USA), anti-Ang2 (ab56301, Abcam), anti-PDGF-B (ab23914, Abcam), anti-PDGF-D (40–2100, Invitrogen, Thermo Fisher Scientific, Waltham, MA, USA), and anti-PlGF (sc-1883, Santa Cruz Biotechnology). After treatment with different hcy concentrations, the eyes were collected from the animals. A lysis buffer was used to extract retinal/choroidal proteins. The protein concentration was standardized, and the proteins were detected and quantified via electrochemiluminescence (Plus-ECL, PerkinElmer, Waltham, MA, USA) and densitometry.

### Immunofluorescence Staining

Immunofluorescence staining was performed on the eye sections of the animals to examine the location and expression of different types of VEGFs, and the eyes were collected following euthanasia and cryostat-sectioned at 5 μm thickness. Each section was mounted on a slide and prepared for immunohistochemical staining. Anti-VEGF and anti-PlGF were used as the primary antibodies, and Alexa 488 (A11055, Molecular Probes, Life Technologies, Grand Island, NY, USA) and Alexa 555 (A31572, Molecular Probes) were used as the secondary antibodies. Isolectin IB4 conjugated Alexa 594 (121413, Molecular Probes, Life Technologies, Grand Island, NY, USA) was used to stain the endothelial cells. Cell nuclei were stained with 4′,6-diamidino-2-phenylindole (DAPI, sc-3598, Santa Cruz Biotechnology). An upright fluorescence microscope (DM2500, Leica) with a cooled Charge-coupled Device (CCD) camera system (CoolSNAP EZ, Roper Scientific, Martinsried, Germany) was used to observe epifluorescence expression of the retinal sections.

### Choroidal capillary sprouting assay

The choroidal capillary sprouting model was adapted from the study of Shao *et al*. with minor modifications^54^. We used both female and male C57BL/6 mice aged 8–12 weeks (BioLASCO Taiwan, Taipei, Taiwan). All animal experiments were conducted in accordance with the Statement for the Use of Animals in Ophthalmic and Vision Research of the Association for Research in Vision and Ophthalmology, and approved by the Institutional Animal Care and Use Committee of Fu-Jen Catholic University. The chorioretinal fragments (retina, RPE layer, and choroid) obtained from the animals were isolated, mounted with Matrigel (Sigma-Aldrich Corp., St Louis, MO, the USA), and seeded in 24-well culture plates. The choroidal tissue was incubated in cell systems complete classic medium (Cat. no.: 420–500; Cell Systems, Kirkland, WA, the USA) with or without hcy (1 mM). The culture medium was changed daily for 5 consecutive days. The area within the circle that connects the outer ends of the choroidal capillary sprouts from each choroidal explant was measured every 24 h for 5 days (120 h). In some preparations, aflibercept (1 mg/mL; Bayer, Leverkusen, Germany) or ranibizumab (0.25 mg/mL; Novartis, Basel, Switzerland) were added to the medium with or without hcy (1 mM) to evaluate their effects. Images were acquired using a stereomicroscope (Leica M165FC, Wetzlar, Germany) and a digital camera (Leica DFC310FX, Wetzlar, Germany) at each time point.

### Statistical Analysis

Data from protein expressions were analyzed using SPSS Statistics, version 20 software (IBM, Armonk, New York, USA), and statistical analysis was performed to determine protein expression differences between the groups. One-way ANOVA with Dunnett’s test or LSD for multiple comparisons was used to determine the significance of the differences between the groups. Differences were considered to be statistically significant when *P* < 0.05.

## Electronic supplementary material

Supplementary figures and table
