# Supplementary material for: Hyperhomocysteinemia Causes Chorioretinal Angiogenesis with Placental Growth Factor Upregulation
Source: Sci Rep. 2018 Oct 25;8:15755. doi: 10.1038/s41598-018-34187-z (PMC6202361; doi:10.1038/s41598-018-34187-z)
Supplement: Supplementary file 1 — Supplementary figures and table [file 41598_2018_34187_MOESM1_ESM.pdf]

Hyperhomocysteinemia Causes Chorioretinal Angiogenesis with Placental Growth Factor Upregulation

Yih-Jing Lee[1,]*, Chia-Ying Ke[1], Ni Tien[1], Po-Kang Lin[2,3,]*

[1] School of Medicine, Fu-Jen Catholic University, Hsinchuang, New Taipei City 24205, Taiwan

[2] Department of Ophthalmology, School of Medicine, National Yang-Ming University, Taipei 11221, Taiwan

[3] Department of Ophthalmology, Taipei Veterans General Hospital, Taipei 11217, Taiwan

Running title: Hyperhomocysteinemia causes choroidal angiogenesis

*Correspondence:

Po-Kang Lin, MD
Department of Ophthalmology, School of Medicine, National Yang-Ming University
No. 155, Section 2, Linong Street, Beitou District, Taipei 11221, TAIWAN
Email: pklin123@hotmail.com

and

Yih-Jing Lee, PhD

School of Medicine, Fu-Jen Catholic University

510 Chungcheng, Hsinchuang, New Taipei City 24205, TAIWAN

Email: yjlee@mail.fju.edu.tw

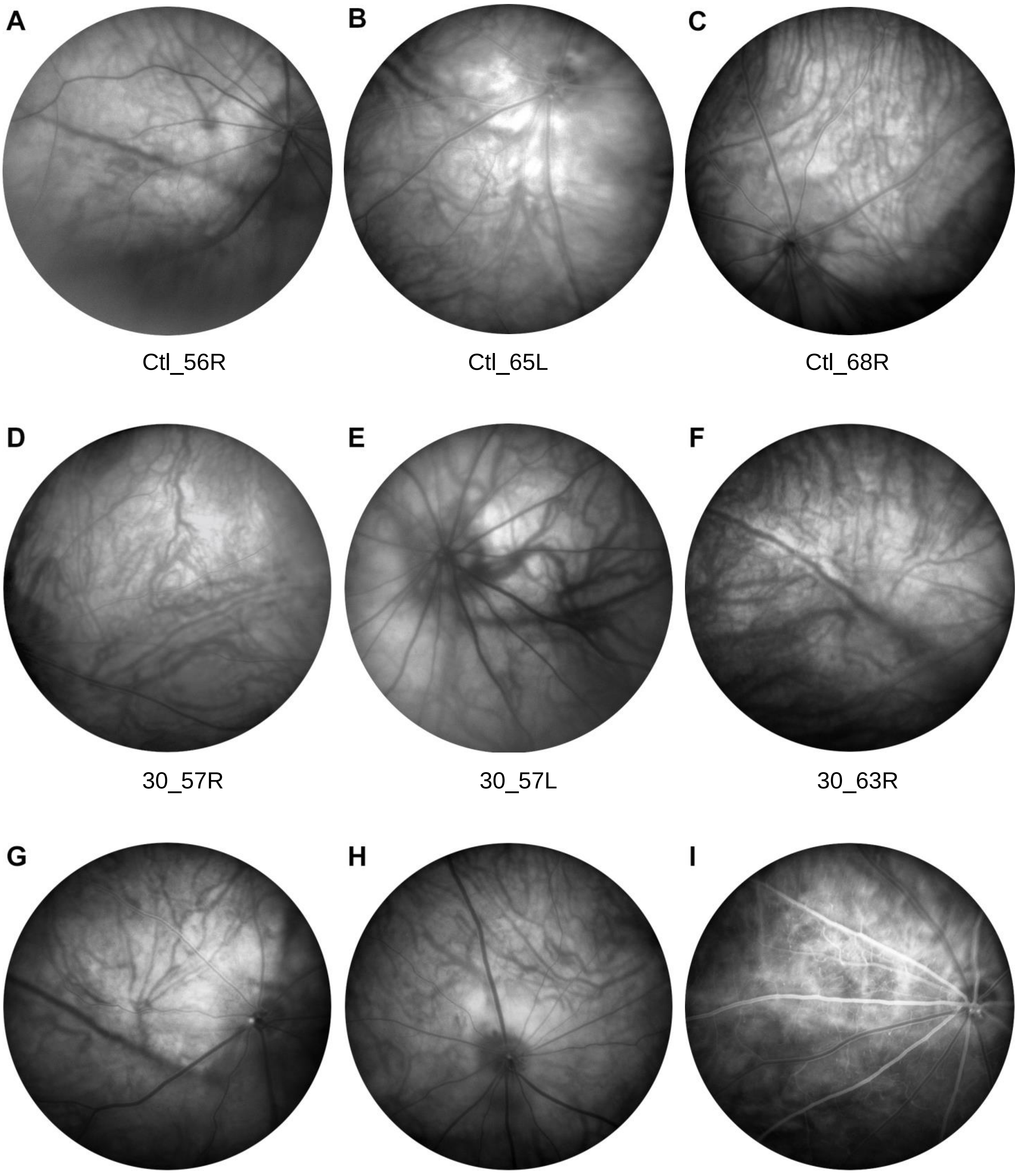
A
B
C
Ctl_56R
Ctl_65L
Ctl_68R
D
E
F
30_57R
30_57L
30_63R
G
H
I
60_66R
60_66L
60_69L

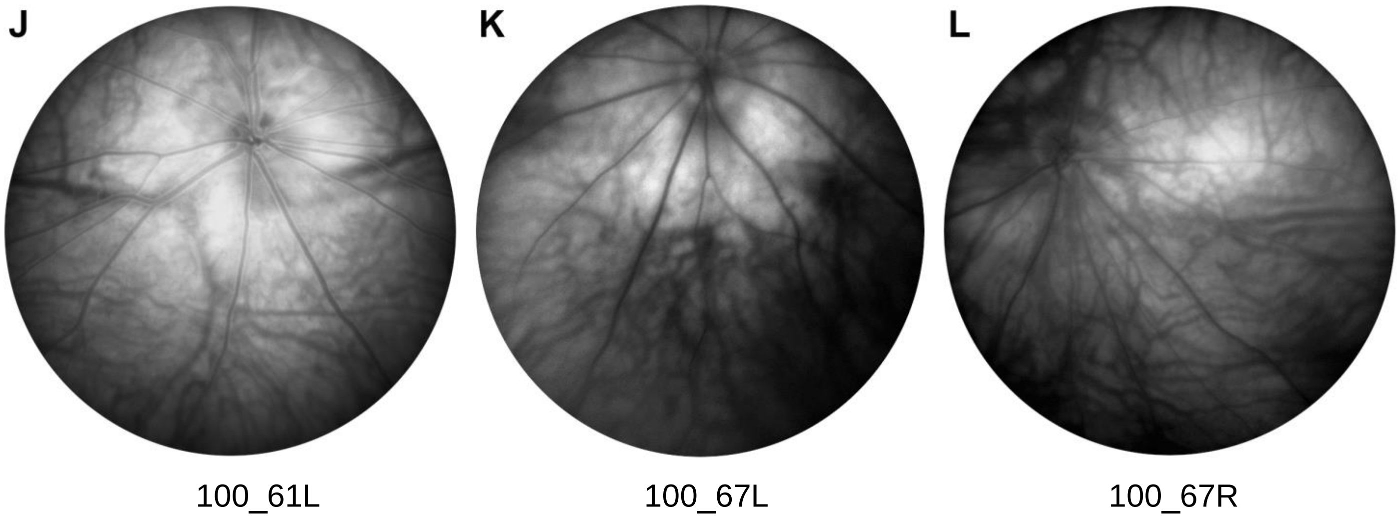

Supplementary Figure 1 Original FAG images used for analysis in table 1.

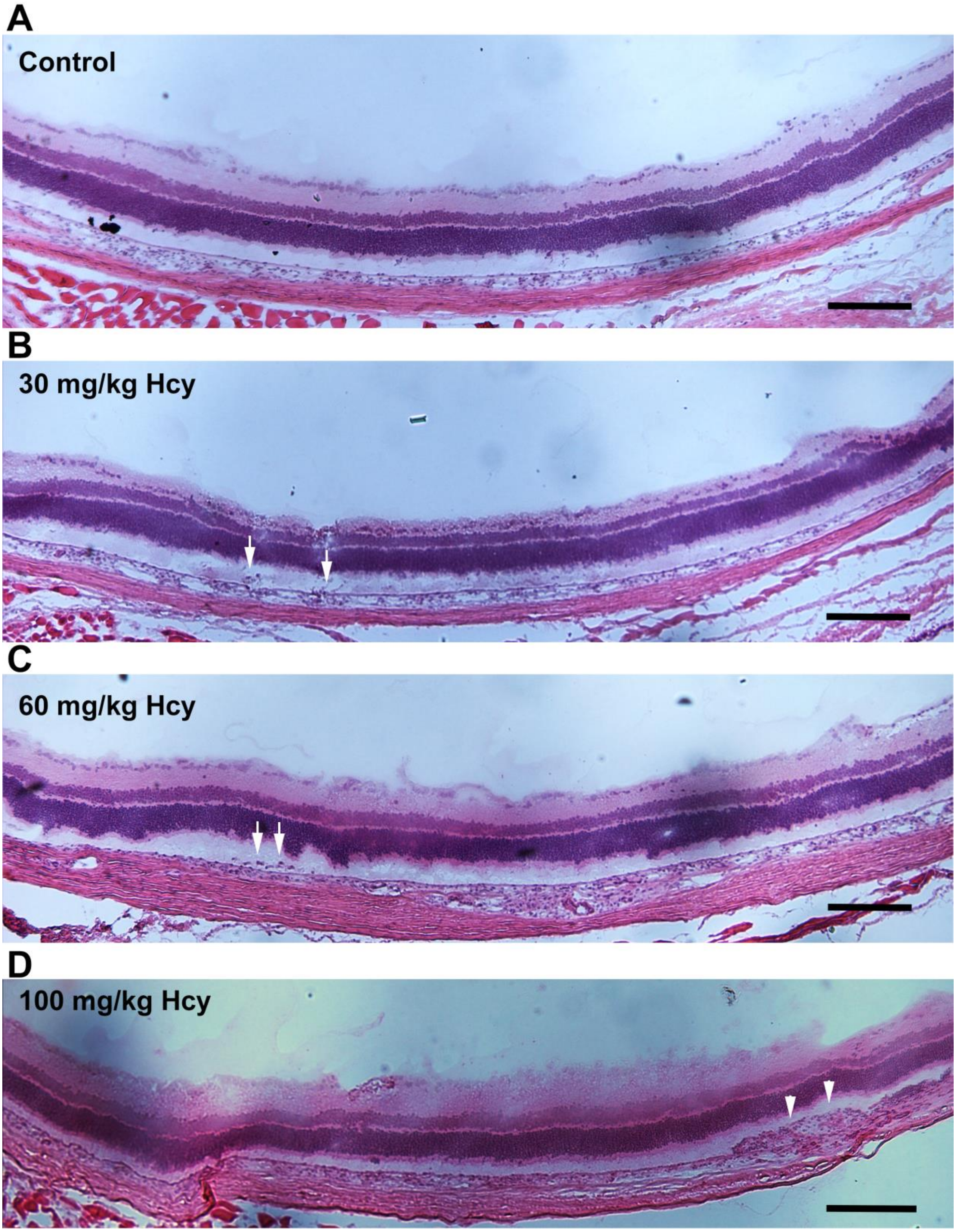

Supplementary Figure 2 Microscopic images of retinas from animals treated with different hcy and control groups, with low power. Eyes were cut into 5-μm-thick sections using a cryostat. H&E staining was applied to observe the histology of the retina. (A) is from control group; (B), (C), and (D) are from 30, 60, and 100 mg/kg hcy treated group. Arrows in (B) and (C) indicate angiogenesis, and arrowheads in (D) indicate cell proliferation. Scale bar = 200 um.

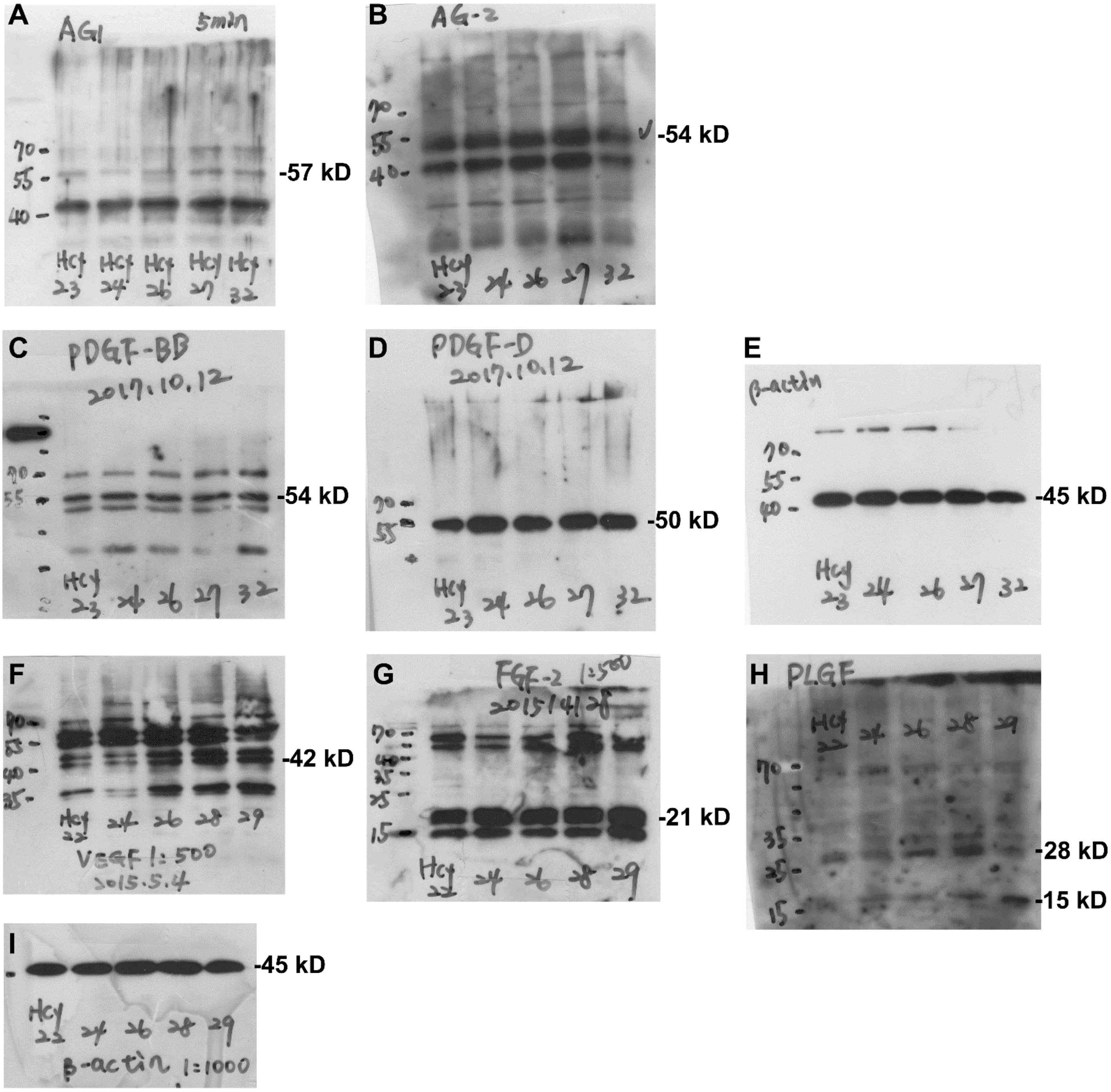

Supplementary Figure 3 Original full-length blots for Figures 4 and 5. (A), (B), (C), (D), and (E) are original blots of Ang1 (A), Ang2 (B), PDGF-B (C), PDGF-D (D), and beta-actin (E) protein expression in figure 4A. (F), (G), (H), and (I) are original blots of VEGF (F), bFGF (G), PlGF (H), and beta-actin (I) protein expression in figure 5A. Molecular weight of target proteins are labeled at the side of the blots. Samples from different preparations are labelled at the bottom of the blots: Hcy22 and Hcy23 are from control group; Hcy24 is from 1 mg/kg-hcy group; Hcy26 is from 10 mg/kg-hcy group; Hcy27 and Hcy28 are from 30 mg/kg-hcy group; Hcy29 and Hcy32 are from 100 mg/kg-hcy group.

Supplementary Table 1 Statistical details for Figures 4 and 5. Data are presented as ratio to protein expression of β-actin and shown as mean ± SEM. One-way ANOVA with Dunnett's test for multiple comparisons was used for the data analysis, * indicates *P<0.05* compared with the control group.

**Hcy** (mg/kg)	**Ang1** (n=5)	**Ang2** (n=5)	**PDGF-B** (n=5)	**PDGF-D** (n=5)	**VEGF** (n=5)	**bFGF** (n=5)	**PlGF dimer** (n=5)	**PlGF monomer** (n=5)	**PlGF total** (n=5)
**0**	0.104 ±0.015	0.280 ±0.067	0.610 ±0.083	1.462 ±0.253	0.144 ±0.034	0.566 ±0.046	0.027 ±0.010	0.012 ±0.007	0.039 ±0.015
**1**	0.069 ± 0.18	0.626 ± 0.138	0.448 ±0.028	0.998 ±0.149	0.095 ±0.024	0.687 ±0.159	0.113 ±0.056	0.007 ±0.004	0.120 ±0.054
**10**	0.083 ±0.011	0.306 ±0.111	0.530 ±0.079	1.038 ±0.292	0.130 ±0.032	0.579 ±0.052	0.040 ±0.014	0.003 ±0.003	0.043 ±0.012
**30**	0.121 ±0.020	0.466 ±0.091	0.686 ±0.205	1.632 ±0.383	0.072 ±0.020	0.537 ±0.120	0.155* ±0.057 ***P*=0.036**	0.027 ±0.023	0.182* ±0.043 ***P*=0.016**
**100**	0.129 ±0.014	0.416 ±0.068	0.478 ±0.061	1.446 ±0.170	0.224 ±0.022	0.577 ±0.055	0.025 ±0.003	0.136* ±0.036 ***P*=0.000**	0.161* ±0.033 ***P*=0.037**
